# Hot Deformation and Constitutive Modeling of TC21 Titanium Alloy

**DOI:** 10.3390/ma15051923

**Published:** 2022-03-04

**Authors:** Sheng-Xian Yi, Zhong-Jiong Yang, Huang-Xin Xie

**Affiliations:** State Key Laboratory of High-Performance Complex Manufacturing, School of Mechanical and Electrical Engineering, Central South University, Changsha 410083, China; shengxian21@126.com (S.-X.Y.); xiehuangxincqu@163.com (H.-X.X.)

**Keywords:** TC21 titanium alloy, hot tensile deformation, deformation mechanism

## Abstract

Titanium alloys are extensively employed in the fabrication of various aviation structural parts, of which the most crucial processing step is hot working. In order to study the high-temperature deformation behavior of the TC21 titanium alloy, high-temperature tensile tests were performed. The results reveal that the flow stress of the material gradually decreases with an increased strain rate, and the stress increases rapidly with an increase in strain during the deformation of the alloy. Following this, flow stress gradually decreases. Flow stress decreases sharply, and the sample fractures when the appearance of necking and microvoids is observed. The Arrhenius and Radial basis function (RBF) neural network constitutive models are established in order to accurately describe the high-temperature deformation behavior of the material. In the modified Arrhenius model, strain rate indexes are expressed as a function of deformation temperature and strain rates; furthermore, the high prediction ability of the model was obtained. For the Radial basis function, the network parameters were obtained using the trial-and-error method. The established models could better forecast the flow stress of materials, and highly accurate results are obtained using the radial basis function model. The relationships between the stress index and the deformation activation energy with strain indicate that the primary deformation mechanism involves grain boundary slip and viscous slip of dislocations. The process of dynamic recrystallization primarily promotes the softening of the material.

## 1. Introduction

Titanium alloys are the best choice in the fabrication of various aviation structural parts (such as landing gears used in aircraft and other structural components of aircraft) as they are characterized by high specific strength, good mechanical properties, and excellent corrosion resistance [1,2]. Titanium alloys crystallize in two crystal structures where the atoms are packed to form HCP and BCC structures. Hot-working can be conducted under conditions of binary (α + β) phase fields to promote mechanical properties of titanium alloy [3]. Moreover, hot forging is considered as the most crucial processing step to refine the microstructure and improve mechanical properties of Ti alloy component [4]. The poor forming ability and narrow forming process parameter range limit the usage of these high-strength titanium alloys.

The process parameters of titanium alloys dictate microstructure evolutions and mechanical property evolution characteristics [5,6]. Qu et al. [7] reported that the softening of Ti-55511 alloy can proceed via two softening pathways. When the initial strain rates ($\dot{\varepsilon }$) increase, dynamic recrystallization (DRX) gradually decreases. It was also observed that the size of grains produced in DRX is negatively correlated with initial $\dot{\varepsilon }$. Liu et al. [8] reported that the process of dynamic recovery (DRV) was primarily involved in the softening of Ti-55511 during deformation in the single-phase region. Dikovits et al. [9] found that the DRX of the Ti-55531 alloy could be observed during the deformation in the β-phase region. The softening of the material could be primarily attributed to the process of DRV. Matsumoto et al. [10] reported high-temperature deformation behavior of single-phase Ti-5Al-5V-5Mo-3Cr titanium alloy, and it was observed that the dynamic precipitation of the α phase affected the flow behavior of the material. Astarita et al. [11] studied the microstructural changing of the TC4 Ti alloy. It was found that complete recrystallization could be realized under these conditions. Fan et al. [12] reported the deformation behavior and the corresponding microstructure of Ti-55531 in the single-phase region, and it was found that $\dot{\varepsilon }$ greatly affected the microstructure. Ning et al. [13,14,15] studied DRV, DRX, and the work-hardening behavior of the TC18 alloy, and the constitutive model of the material was established. The DRV and DRX that occurred in the α phase significantly affected the high-temperature deformation behavior of titanium alloys. Song et al. [16] investigated the dynamic spheroidization of the dual-phase titanium alloy, and they reported that a small amount of the equiaxed α phase could be formed under high temperature, high $\dot{\varepsilon }$, and large extents of deformation. Mirone and Barbagallo [17] reported metal sensitivity to strain, $\dot{\varepsilon }$, and temperature during necking initiation and hardening in dynamic testing.

Numerous constitutive models have been developed to accurately express high-temperature deformation behaviors of alloys. Peng et al. [18] developed a high-temperature constitutive model to show the behavior of the TC4-DT titanium alloy. Kotkunde et al. [19] developed four different constitutive models to describe the deformation of the TC4 alloy. It was found that the MTS constitutive model was characterized by good prediction accuracy, and it could be effectively used to depict high-temperature flow behaviors of the materials. Bai et al. [20] studied the primary softening mechanism associated with the softening of the TC4 alloy. The effects of α-phase spheroidization, dislocation density, temperature rise, and phase transition on the flow stress of materials were analyzed, and a unified model was built. Machine learning is an important part of artificial intelligence. By conducting machine learning, computers can continuously absorb new knowledge and reconstruct existing knowledge in order to constantly improve their properties and complete data updates [21,22]. Wang et al. [23] used the machine learning technique to predict the mechanical properties of steel, and the microstructure of the alloy was inversely deduced based on mechanical properties. Mangala and Holm [24] predicted the hot spot stress of the alloy. Shen et al. [25] developed the constitutive model to study the Ti-4Al-3V-2Mo-2Fe titanium alloy. Shen et al. [26] studied a metamodeling method to simulate the constitutive relationship of the TC6 titanium alloy. Abueidda et al. [27] used deep learning to model the plasticity and thermo-viscoplasticity of titanium alloy.

The TC21 titanium alloy can be reinforced through heat treatment. Although researchers from various countries have widely studied the structure evolution of titanium alloys, they have rarely studied the thermal deformation behavior of TC21 alloy. To investigate the deformation behavior of TC21 titanium alloy, high-temperature tensile tests were conducted. We aimed to analyze the influence of deformation process parameters on the deformation behavior of alloys and establish an accurate constitutive model.

## 2. Material and Methods

**Figure 1:**
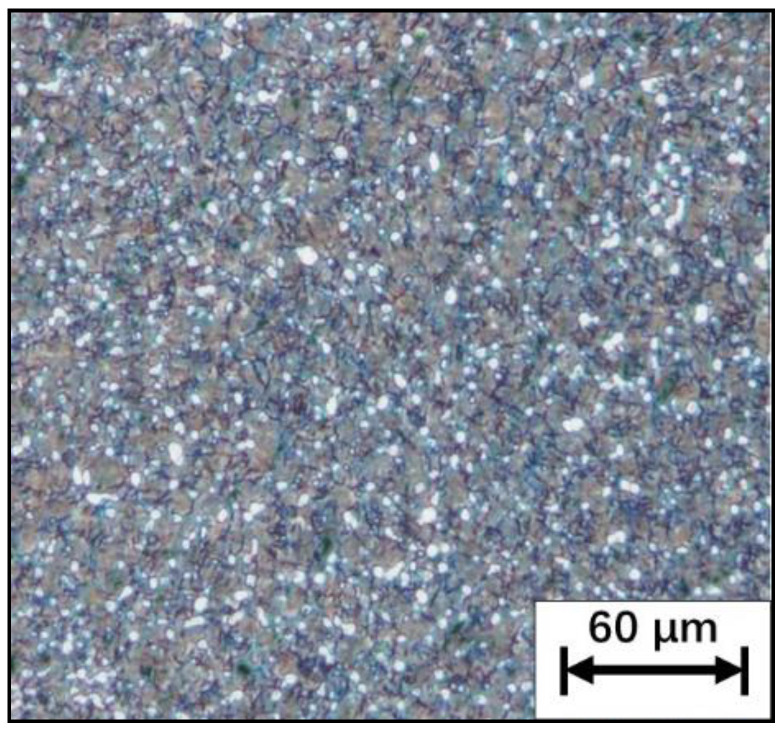
Initial microstructure of the material.

**Figure 2:**
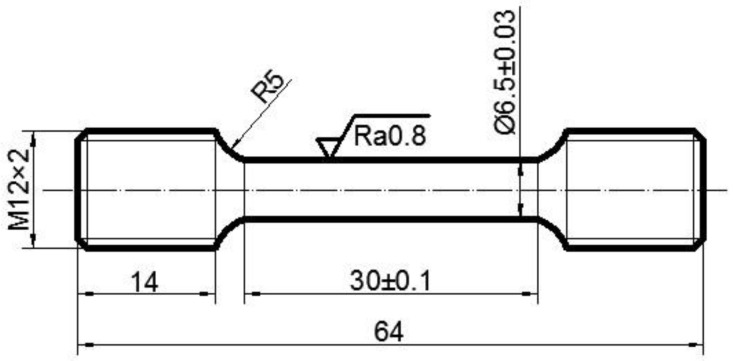
Shape of the high temperature tensile specimen (unit: mm).

The nominal composition of the TC21 alloy used in the experiment is (wt.%) Ti-6Al-2Zr-2Sn-2Mo-1.5Cr-2Nb, which is a typical Ti-Al-Sn-Zr-Mo-Cr-Nb(-Ni-Si) alloy. The β transition temperature of the TC21 alloy is approximately 975 °C. The cast TC21 alloy is forged at the beta region first and then deformed at the alpha + beta region. The forged alloy is used in this work. The initial microstructure of the alloy was characterized using Olympus DSX500 optical microscope. The initial microstructure (α + β bimodal microstructure) of the material is shown in Figure 1. Moreover, the size of the specimen is shown in Figure 2.

The test is conducted using an Instron 3369 high-temperature testing machine. The temperature control accuracy of the testing machine is ±1 °C, the force control accuracy is ±0.1 MPa, and the displacement control accuracy is ±1 μm. At the beginning of the experiment, the sample was heated to the specified temperature (820 °C, 870 °C, and 920 °C) at an initial heating rate of 20 °C/min. The temperature was increased slowly to the given temperature when the temperature is close to the given value due to the PID control strategy of the equipment. The entire heating time is about 1 h. Then, the samples were cooled down to 20 °C in the heating furnace after the tests. This temperature was maintained for 5 min to guarantee the generation of a uniform temperature. Following this, the sample was stretched at a constant stretching speed (0.01, 0.1, or 0.28 s^−1^). Subsequently, the sample was cooled to room temperature at a cooling rate of 50 °C/min. During the experiment, the temperature, force, and strain were collected simultaneously.

Due to the measuring range limitation of the strain extensometer, the strains were collected using the external extensometer first. When the strain reached the maximum measuring range of the external physical extensometer, the extensometer was removed, and the strains were collected using in-built extensometers of the Instron machine. In order to gain accurate stress–strain curves, the tests were repeated two times at each deformation condition, and the average values were shown in the true stress strain curves below.

## 3. Results and Discussion

### 3.1. High-Temperature Deformation Behavior of the Materials

**Figure 3:**
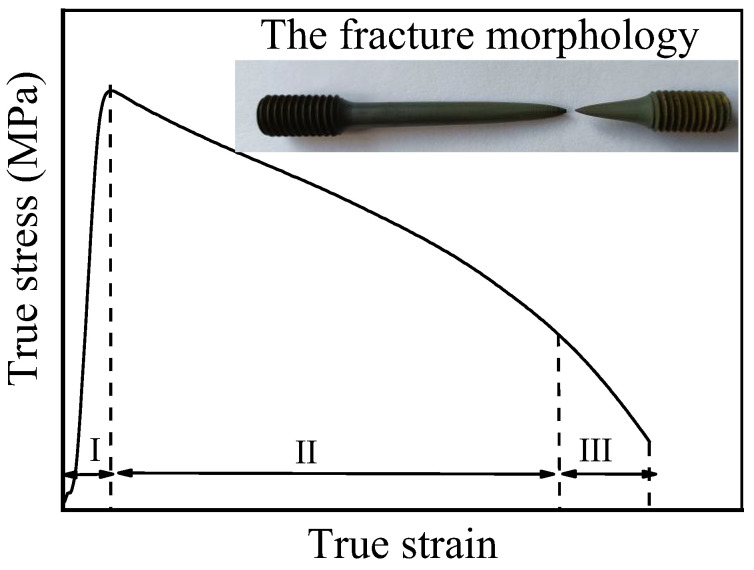
High temperature deformation characteristics.

Figure 3 presents high-temperature deformation characteristics of the TC21 titanium alloy. As shown in Figure 3, the high-temperature deformation process of TC21 ALLOY can be divided into three stages [28]. With an increase in deformation in the first stage, i.e., the work hardening stage (Stage I), the flow stress of the material increases rapidly, and the peak values appear during the stable deformation stage (Stage II); meanwhile, DRV and DRX occur inside the material. As the strain increases, the flow stress gradually decreases, indicating uniform flow characteristics. In the third stage, i.e., the metastable flow stage (Stage III), as the process of stretching progresses, the inner part of the material shrinks, and microvoids are generated. As the load-bearing capacity of the material decreases gradually, the flow stress decreases rapidly, resulting in material fracture (Figure 3).

**Figure 4:**
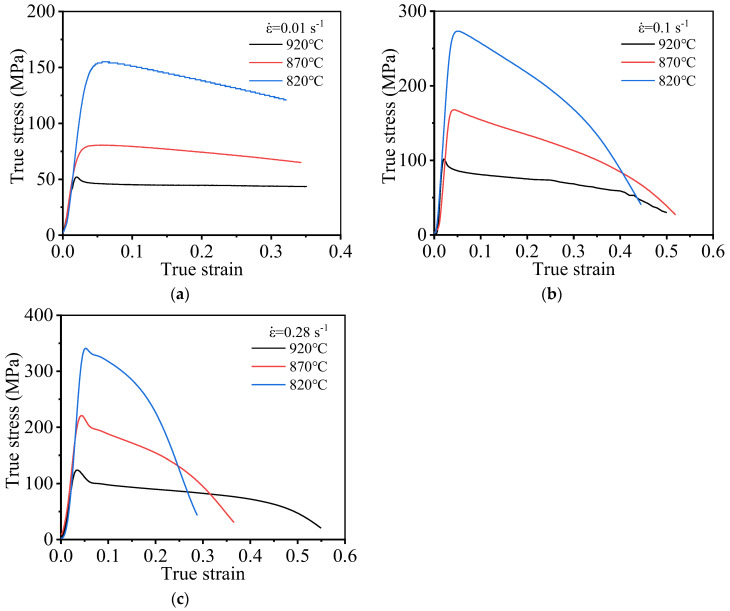
True stress–true strain curve under different deformation strain rates: (**a**) 0.01 s^−1^; (**b**) 0.1 s^−1^; (**c**) 0.28 s^−1^.

Figure 4 presents the stress–strain curves generated during high-temperature stretching at varying temperatures. As shown in Figure 4, the high-temperature deformation behavior of the TC21 titanium alloy shows the obvious dynamic softening behavior. The flow stress increases at first, and it gradually decreases as the strain increases after reaching its peak value. The accumulation layer corresponding to the titanium alloy is high, and dislocations are prone to cross slip and climbing; therefore, the deformation resistance of the material decreases. As the extent of deformation increases, the distortion energy of the material increases. Under these conditions, the initiation of DRV and DRX can potentially occur, resulting in the softening of the material. Therefore, as deformation progresses, the work hardening phenomenon gradually weakens, and the flow stress of the material gradually decreases.

An analysis of the flow stress curves generated at varying temperatures reveals that the *T* significantly influences the high-temperature deformation behavior of TC21 alloy. Moreover, by increasing deformation temperature (*T*), flow stress decreases, which is attributed to the thermal activation of dislocation. The ability of the dislocation to move increases as temperature increases. Dislocation slip or dislocation climb can potentially occur under these conditions, resulting in the plastic deformation of the material, and the nucleation rate and the grains growth rate significantly increase with increased temperature. Under these conditions, the extent of DRV realized increases, increasing the extent of the softening of the material. This renders the material susceptible to plastic deformation.

The high-temperature tensile flow stress of TC21 titanium alloy is affected by $\dot{\varepsilon }$ (Figure 4). When $\dot{\varepsilon }$ increases, the flow stress under the same strain gradually grows. This can be attributed to the fact that the rate of dislocation proliferation (attributable to work hardening) is higher than the rate of dislocation annihilation (attributable to DRV/DRX). Grain nucleation can be readily initiated during the process of DRX under conditions of a high dislocation density. The nucleus associated with the DRX process does not have enough time to grow due to the short deformation time and high strain rate; thus, dislocation multiplication (attributable to work hardening) cannot be avoided. Therefore, the higher the strain rate, the larger the dislocation density and the flow stress.

**Figure 5:**
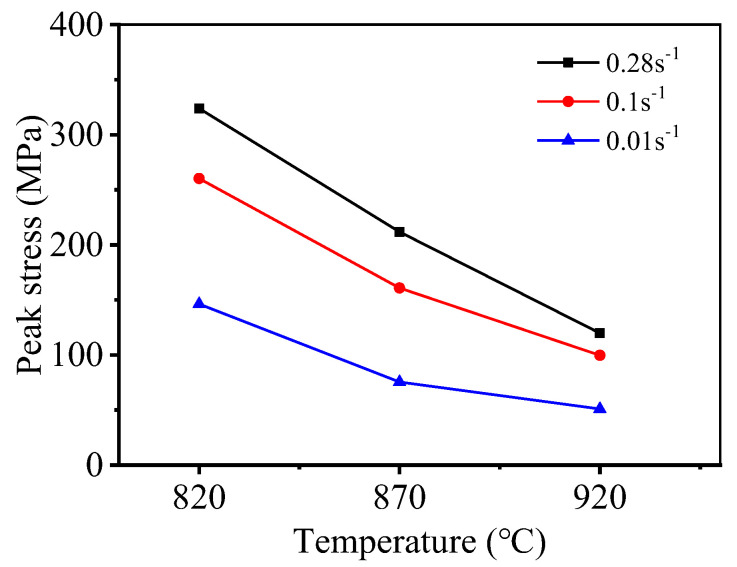
The influence of deformation process conditions on alloy peak stress.

Figure 5 presents the peak stress of TC21 alloy under varying deformation conditions. Figure 5 reveals that the deformation conditions that significantly affect the peak stress of TC21 titanium alloy. Peak stress gradually decreases as *T* increases and $\dot{\varepsilon }$ decreases, and the rate of dislocation proliferation and dislocation density decreases, and the peak stress of the material decreases.

### 3.2. Constitutive Description of the TC21 Alloy

In general, the flow stress generated during the deformation process can be expressed as a function of *T*(K), $\dot{\varepsilon }$, and strain. Arrhenius, Johnson–Cook, and Rusinek-Klepaczko models are the commonly used constitutive models. The Arrhenius-type constitutive model is often used to describe the high-temperature deformation behavior of materials as the model is characterized by a few parameters. The Arrhenius-type constitutive model can be expressed by Equation (1) as follows.

(1) $$\dot{\varepsilon }=AF(\sigma )\exp(-\frac{Q}{RT})$$

According to the different stress levels, there are different expressions of *F*(*σ*):

(2) $$F(\sigma )=\{\begin{array}{ll} \sigma^{n} & \alpha \sigma <0.8\\ \exp(\beta \sigma ) & \alpha \sigma >1.2\\ {\left[\sinh(\alpha \sigma )\right]}^{m} & \mathrm{all}\;\sigma \end{array}$$

where α=β/n *σ* is flow stress (MPa), R is the ideal gas constant (8.31 J/(mol⋅K)), *Q* is the deformation activation energy (kJ/mol), and A, β, n, and m denote material constants.

The influence of the deformation process parameters ($\dot{\varepsilon },\;T$) on flow stress is usually expressed by the temperature-compensated strain rate factor *Z*, as shown below.

(3) $$Z=\dot{\varepsilon }\exp(\frac{Q}{RT})$$

According to the definition of hyperbolic sine and Equation (3), flow stress can be expressed as a function of the parameter *Z* as follows.

(4) $$\sigma =\frac{1}{\alpha }\ln\left\{{\left(\frac{Z}{A}\right)}^{\frac{1}{m}}+{\left[{\left(\frac{Z}{A}\right)}^{\frac{2}{m}}+1\right]}^{\frac{1}{2}}\right\}$$

Taking the value of true strain as 0.1 (representative example), the method of obtaining the value of each parameter is illustrated. Under low stress (ασ<0.8) and high stress (ασ>1.2) conditions, the expression represented in Equation (2) is substituted into Equation (1). When the deformation activation energy *Q* is independent of *T*, the following relations can be obtained.

(5) $$\dot{\varepsilon }=B\sigma^{n}$$

(6) $$\dot{\varepsilon }=\mathrm{Cexp}(\beta \sigma )$$

Logarithms of Equations (5) and (6) are taken to obtain Equations (7) and (8), respectively. The equations are expressed as follows.

(7) $$\ln\sigma =\frac{1}{n}\ln\dot{\varepsilon }-\frac{1}{n}\ln B$$

(8) $$\sigma =\frac{1}{\beta }\ln\dot{\varepsilon }-\frac{1}{\beta }\ln C$$

**Figure 6:**
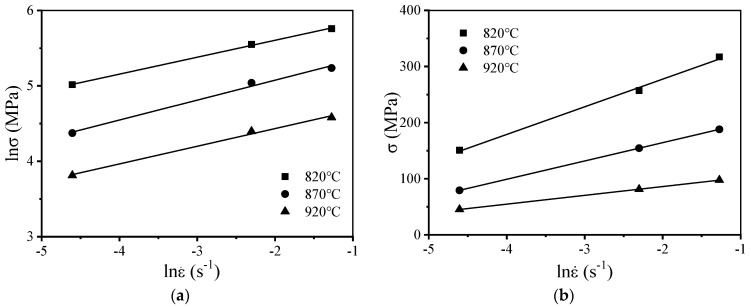
Relationship between stress and strain rate: (**a**) $\ln\sigma \sim \ln\dot{\varepsilon }$; (**b**) $\sigma \sim \ln\dot{\varepsilon }$.

The values of stress and strain were substituted into Equations (7) and (8), considering that strain is 0.1. The linear relationship obtained under these conditions is shown in Figure 6. $\ln\sigma \sim \ln\dot{\varepsilon }$ and $\sigma \sim \ln\dot{\varepsilon }$ in Figure 6 exhibit a linear relationship. The slope of the line is obtained following the least square method. Following this, the average value of the slope is calculated, and its reciprocal value is determined. The value of n is 4.1689, the value of β is 0.03824 MPa^−1^, and the value of α is 0.09173.

For all stress states, Equation (9) can be obtained.

(9) $$\dot{\varepsilon }=A{\left[\sinh(\alpha \sigma )\right]}^{m}\exp(-\frac{Q}{RT})$$

Equation (10) can be obtained by taking the logarithm of both sides of Equation (9). Equation (10) is expressed as follows.

(10) $$\ln[\sinh(\alpha \sigma )]=\frac{\ln\dot{\varepsilon }}{m}+\frac{Q}{\mathrm{mR}T}-\frac{\ln A}{m}$$

**Figure 7:**
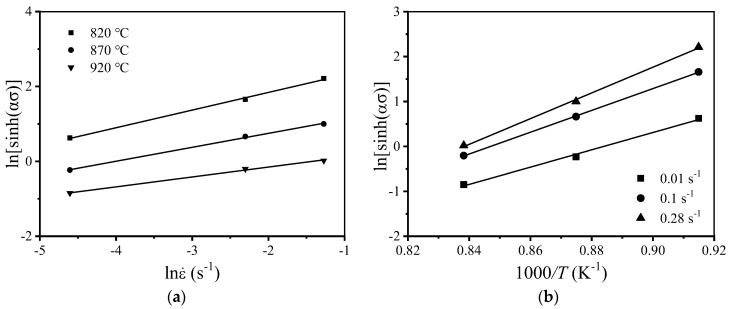
Relationship between stress and strain rate and temperature: (**a**) $\ln[\sinh(\alpha \sigma )]-\ln\dot{\varepsilon }$; (**b**) ln[sinh(ασ)]-1/T.

The relationship between $\ln[\sinh(\alpha \sigma )]-\ln\dot{\varepsilon }$ and ln[sinh(ασ)]-1/T at strain value 0.1 is presented in Equation (10). The linear relationship between $\ln[\sinh(\alpha \sigma )]-\ln\dot{\varepsilon }$ and ln[sinh(ασ)]-1/T is obtained. The slope of the straight line of $\ln[\sinh(\alpha \sigma )]-\ln\dot{\varepsilon }$ is the reciprocal of n, and the value of m is found to be 2.8632. The value of m is used to obtain a *Q* value, 572.8915 kJ/mol. The intercept of the straight line presented in Figure 7a can be expressed as $\frac{Q}{\mathrm{mR}T}-\frac{\ln A}{m}$. The value of A can be obtained by substituting the calculated m and *Q* values. The value of lnA is found to be 55.9645.

**Figure 8:**
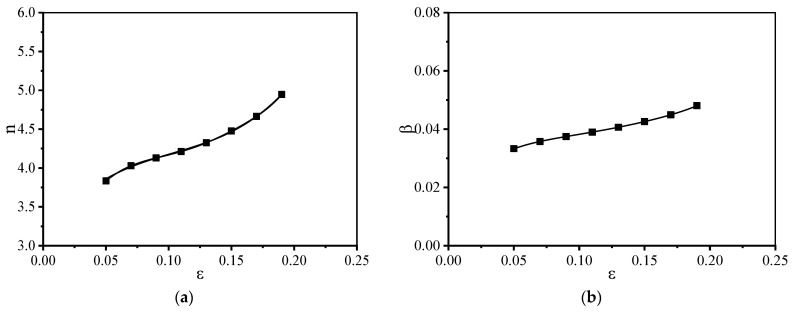

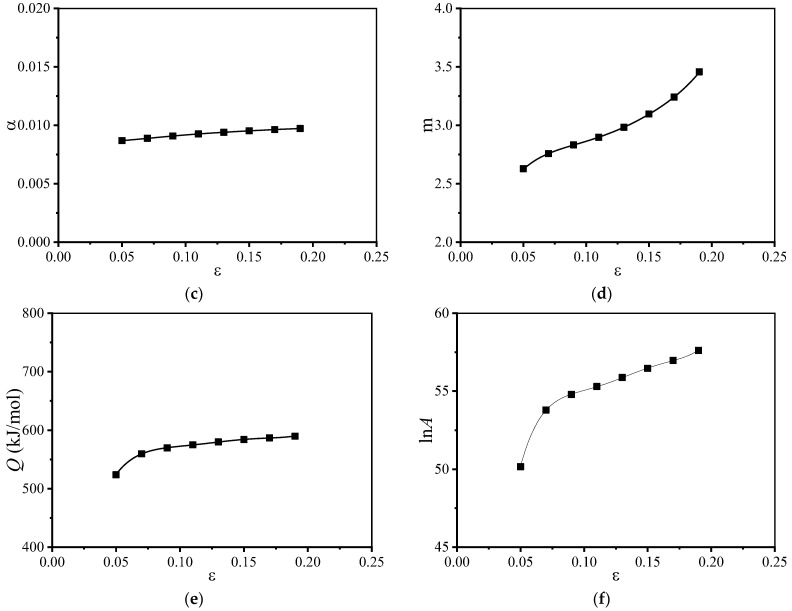
The relationship between the material constant and the true strain: (**a**) $n^{\prime }$; (**b**) β; (**c**) α; (**d**) n; (**e**) Q; (**f**) lnA.

The method mentioned above is followed to select the strain values (interval: 0.02) in the range of 0.05–0.2 to calculate the material parameter values (*Q*, A, β, α, n, and m) associated with the constitutive model. The relationship between material constant and true strain is shown in Figure 8. Material constants and strains could be expressed by a polynomial as follows (Equation (11)).

(11) $$\left[\begin{array}{l} n\\ m\\ \alpha \\ \beta \\ \begin{array}{l} Q\\ \ln A \end{array} \end{array}\right]=\left[\begin{array}{llllll} 1.42 & 101.58 & -1577.44 & 12383.86 & -47468.00 & 73048.79\\ 1.13 & 60.08 & -954.35 & 7503.13 & -28871.72 & 44989.90\\ 0.0090 & -0.031 & 0.77 & -6.77 & 27.28 & -42.18\\ 0.014 & 0.75 & -10.51 & 80.25 & -303.88 & 471.46\\ 26.61 & 21356.05 & -335876.36 & 2623340 & -10048800 & 15078200\\ -2.13 & 2256.35 & -35681.32 & 279175.49 & -1068400 & 1601900 \end{array}\right]\left[\begin{array}{l} 1\\ \varepsilon \\ \varepsilon^{2}\\ \varepsilon^{3}\\ \varepsilon^{4}\\ \varepsilon^{5} \end{array}\right]$$

**Figure 9:**
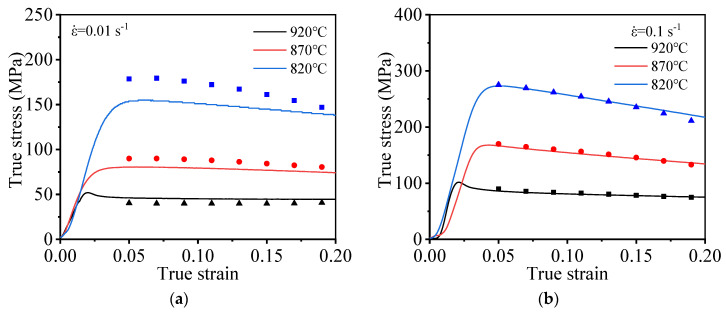

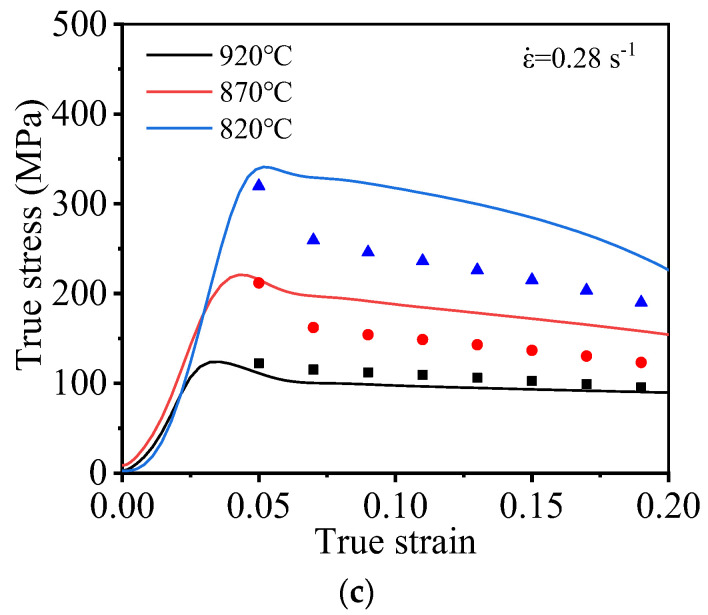
Experimental and predicted values at different deformation conditions: (**a**) 0.01 s^−1^; (**b**) 0.1 s^−1^; (**c**) 0.28 s^−1^.

The experimentally obtained values and the model-predicted values obtained under conditions of varying temperatures and $\dot{\varepsilon }$ were compared to verify the accuracy of the model. The results are presented in Figure 9. When $\dot{\varepsilon }$ is 0.1 s^−1^, a high prediction accuracy is realized, while the prediction accuracy is unacceptable at other strain rates. Therefore, the model does not fully account for the effect of the strain rate, and further modification is needed to accurately describe the behavior of the materials.

The temperature-compensated $\dot{\varepsilon }$ factor *Z* is corrected as follows.

(12) $$Z={\dot{\varepsilon }}^{p(ln\dot{\varepsilon },\;T)}\exp(\frac{Q}{RT})$$

The *p* values obtained under different deformation conditions are shown in Table 1.

**Figure 10:**
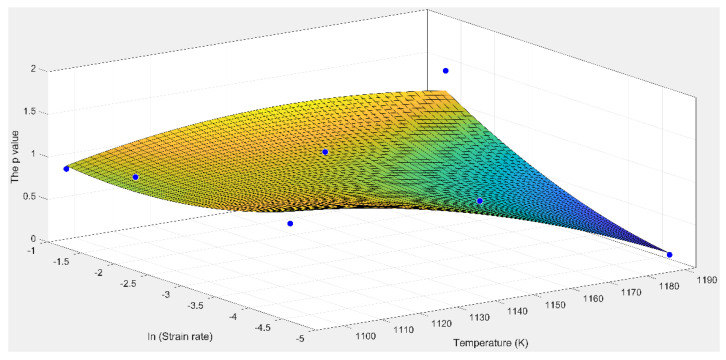
The relationship between parameter *p* and *T* and $\dot{\varepsilon }$.

**Figure 11:**
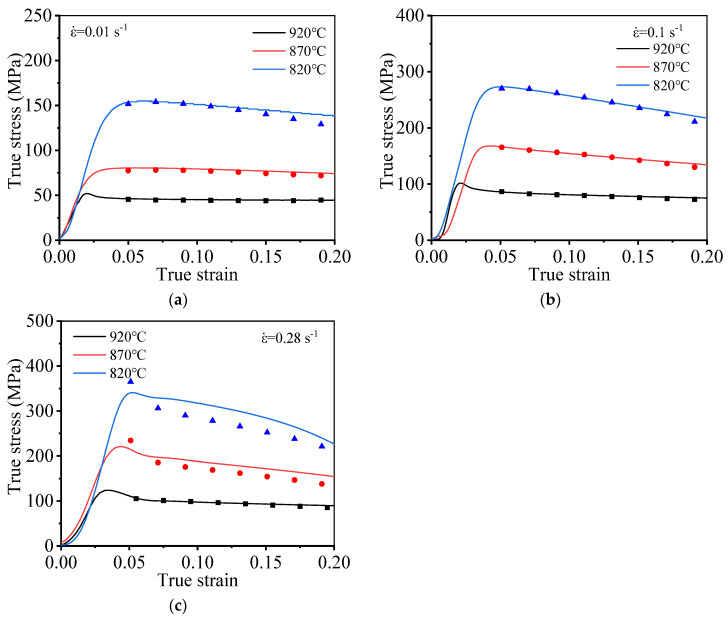
Experimental and predicted values at different deformation conditions: (**a**) 0.01 s^−1^; (**b**) 0.1 s^−1^; (**c**) 0.28 s^−^^1^.

The relationship between *p* and the *T* and $\dot{\varepsilon }$ is obtained by following the locally weighted smooth quadratic regression method, as shown in Figure 10. Experimentally obtained and predicted values of flow stress (obtained under varying deformation conditions) are presented in Figure 11. Figure 11 reveals that the modified predicted flow stress value is in good agreement with the experimentally obtained value. This indicates that the model developed by us can accurately describe the deformation behavior of the alloy. 

### 3.3. Radial Basis Function (RBF) Neural Network Model

**Figure 12:**
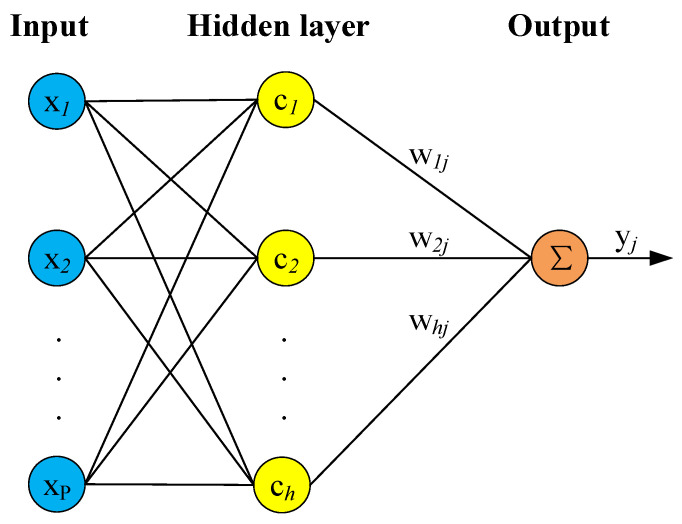
Schematic diagram of radial basis neural network.

The development of data technology has resulted in the establishment and development of data-driven machine learning constitutive models that help in the development of the material constitutive theory. The structure of the RBF neural network is similar to that of the multilayer forward network, and it includes three layers. The nodes associated with the input layer transmit input signals to the hidden layer, and the nodes related to the hidden layer are made up of radical action functions (such as the Gaussian functions). The nodes corresponding to the output layer can usually be represented by simple linear functions. The action function (basic function) in the hidden layer node locally responds to the input signal. The schematic diagram of the RBF neural network is shown in Figure 12.

The activation function of the RBF neural network can be expressed as follows:

(13) $$R(x_{p}-c_{i})=\exp\left(-\frac{1}{2\sigma_{i}^{2}}{\left\|x_{p}-c_{i}\right\|}^{2}\right)\;i=1,2,\cdots ,h\;p=1,2,\cdots ,P$$

where *x_p_* is the *p*-th input sample, *c_i_* is the *i*-th center point, *h* is the number of nodes in the hidden layer, and σ is the expansion coefficient (determined based on the distribution of the data center). The variance is determined as follows to avoid the generation of a flat or significantly sharp RBF.

(14) $$\begin{array}{cc} \sigma_{i}=\frac{c_{\max}}{\sqrt{2h}} & i \end{array}=1,2,\cdots ,h$$

The output of the RBF neural network can be expressed as follows:

(15) $$\begin{array}{cc} \begin{array}{cc} y_{j}=\sum_{i=1}^{h}w_{ij}\exp\left(-\frac{1}{2\sigma^{2}}{\left\|x_{p}-c_{i}\right\|}^{2}\right) & j=1,2, \end{array}\cdots ,n & i=1,2, \end{array}\cdots ,h$$

where n is the number of output samples or classification, and *w_ij_* is the weight between the *i*-th hidden layer and the *j*-th output layer node. 

Appropriate structural and parameter designs should be considered to construct the RBF neural network. The structural design considers the number of hidden layers b and the number of nodes h presented in each hidden layer. The parameter design takes into consideration the data center c of the basis function, the extended parameter σ, and the weight w of the output node. The training error corresponding to the RBF neural network can be expressed as follows:

(16) $$F=\frac{1}{P}\sum_{1}^{n}{\left\|d_{j}-y_{j}\right\|}^{2}$$

where *d_j_* is the actual output value, and *F* is the fitness of the optimization model. The process of obtaining (b*, h*, c*, σ*, and w*) using the optimization algorithm is the training process of the network.

(17) $$\left(b*,h*,c*,\sigma *,w*\right)=\underset{b,h,c,\sigma ,w}{\arg\min}F$$

**Figure 13:**
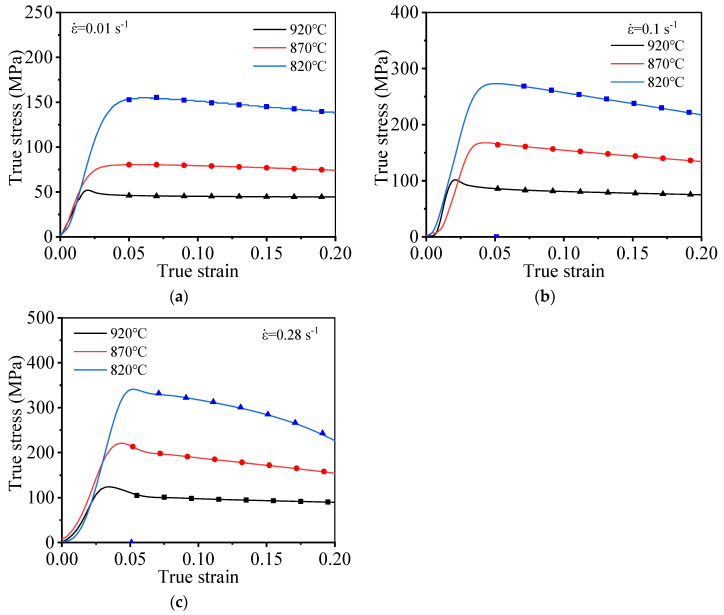
Experimental and predicted values at different deformation conditions: (**a**) 0.01 s^−1^; (**b**) 0.1 s^−1^; (**c**) 0.28 s^−1^.

To obtain the proper network parameters, the number of the hidden layers, the nodes in each hidden layer, and the expansion coefficient are adjusted many times using the trial-and-error method. When the errors between the experimental and predicted values are acceptable, i.e., the correlation coefficient is larger than 0.99, the corresponding network parameters are obtained. Therefore, an RBF neural network model (radial basis expansion speed: 0.0.1) based on the above network model is established. The model consists of two hidden layers, and the number of nodes in each hidden layer is 40. The expansion coefficient is 0.01. The prediction accuracy of the model is shown in Figure 13. Figure 13 indicates that the predicted value is in good agreement with the experimentally obtained value, and the prediction accuracy is higher than the prediction accuracy obtained using the Arrhenius constitutive model. This indicated that the developed model could precisely depict the deformation behavior of the alloy.

### 3.4. High-Temperature Deformation Mechanism Associated with TC18

Stress index m is often used to analyze the primary deformation mechanism associated with a material subjected to thermal deformation. When m is close to 2, the predominant deformation mechanism involves grain boundary slip. When the value of m is 3, the primary deformation mechanism involves dislocation viscous slip. When m is in the range of 4–6, the process of deformation primarily involves the process of dislocation climbing. As shown in Figure 8d, within the range of experimental temperature and strain rate, the value of the stress index n of the material lies in the range of 2–4. Therefore, the process of deformation primarily involves the processes of grain boundary slip and dislocation viscous slip.

The magnitude of the deformation activation energy is influenced by the deformation mechanism [29,30]. The extent of DRV realized is influenced by cross-slip and climb of dislocations. Diffuse softening is observed as the deformation activation energy is approximated to the self-diffusion activation energy of the material. The process of recrystallization is accompanied by the formation of nondistortion subcrystals and the migration of the large-angle grain boundary. The activation energy of the deformation process is greater than that associated with the self-diffusion of the material. As shown in Figure 8e, the activation energy associated with material deformation gradually increases with increased deformation. Figure 8e reveals that the deformation activation energy of materials is in the range of 500–700 kJ/mol. In general, the self-diffused activation energies corresponding to α titanium and β titanium are 169 kJ/mol and 153 kJ/mol [31], respectively. The deformation activation energy is significantly higher than the self-diffusion activation energy of the material. It indicates the extent of DRX and the softening effect are continuously improved [32].

## 4. Conclusions

The high-temperature deformation behavior of the TC21 titanium alloy has been studied by conducting high-temperature tensile tests. The conclusions made have been presented as follows:

(1) The DRX process of the TC21 titanium alloy during high-temperature deformation is observed. The flow stress of the material initially increases and then decreases gradually as the strain increases, which results from the effect of DRX. The gradual decrease can be attributed to the appearance of necking and micropores, which results in material fracture.
(2) The high-temperature rheological behavior of the TC21 titanium alloy is influenced by *T* and $\dot{\varepsilon }$. As $\dot{\varepsilon }$ increases, the flow stress of materials gradually increases. When *T* increases, flow stress gradually decreases.
(3) The Arrhenius and RBF neural network constitutive models, which can accurately describe the deformation process of materials, have been built and the models can well forecast the flow stress of materials at high temperatures. The results obtained from the RBF neural network are more accurate than those obtained using the Arrhenius model.
(4) The relationships between the stress index and the deformation activation energy with strain indicate that the processes of grain boundary slip and viscous slip of dislocations result in material deformation. DRX primarily results in the softening of the material.

## Figures and Tables

**Table 1:** *p*-Value obtained under different deformation conditions.

Temperature (°C)	Strain Rate (s^−1^)	Parameter *p* Value
820	0.01	1.15
	0.1	1.05
	0.28	0.05
870	0.01	1.1
	0.1	1.03
	0.28	0.45
920	0.01	0.93
	0.1	1.07
	0.28	1.35

## Data Availability

Not applicable.
